# Glucocorticoids Combined with Methotrexate in the Treatment of Psoriasis Merged with Bullous Pemphigoid: A Case Report and Literature Review

**DOI:** 10.1002/ccr3.72173

**Published:** 2026-02-28

**Authors:** Weimin Ma, Cheng Li, Haijun Miao

**Affiliations:** ^1^ Department of Dermatology The 940th Hospital of Joint Logistic Support Force of Chinese PLA Lanzhou Gansu Province China

**Keywords:** bullous pemphigoid, glucocorticoids, methotrexate, psoriasis

## Abstract

For psoriatic patients who develop bullous pemphigoid, a rare yet serious complication, prompt initiation of combined glucocorticoid and methotrexate provides rapid disease control and sustained remission. This regimen serves as an effective steroid‐sparing strategy, though long‐term monitoring remains essential to manage recurrence risks during steroid taper.

## 1 Case History

**FIGURE 1:**
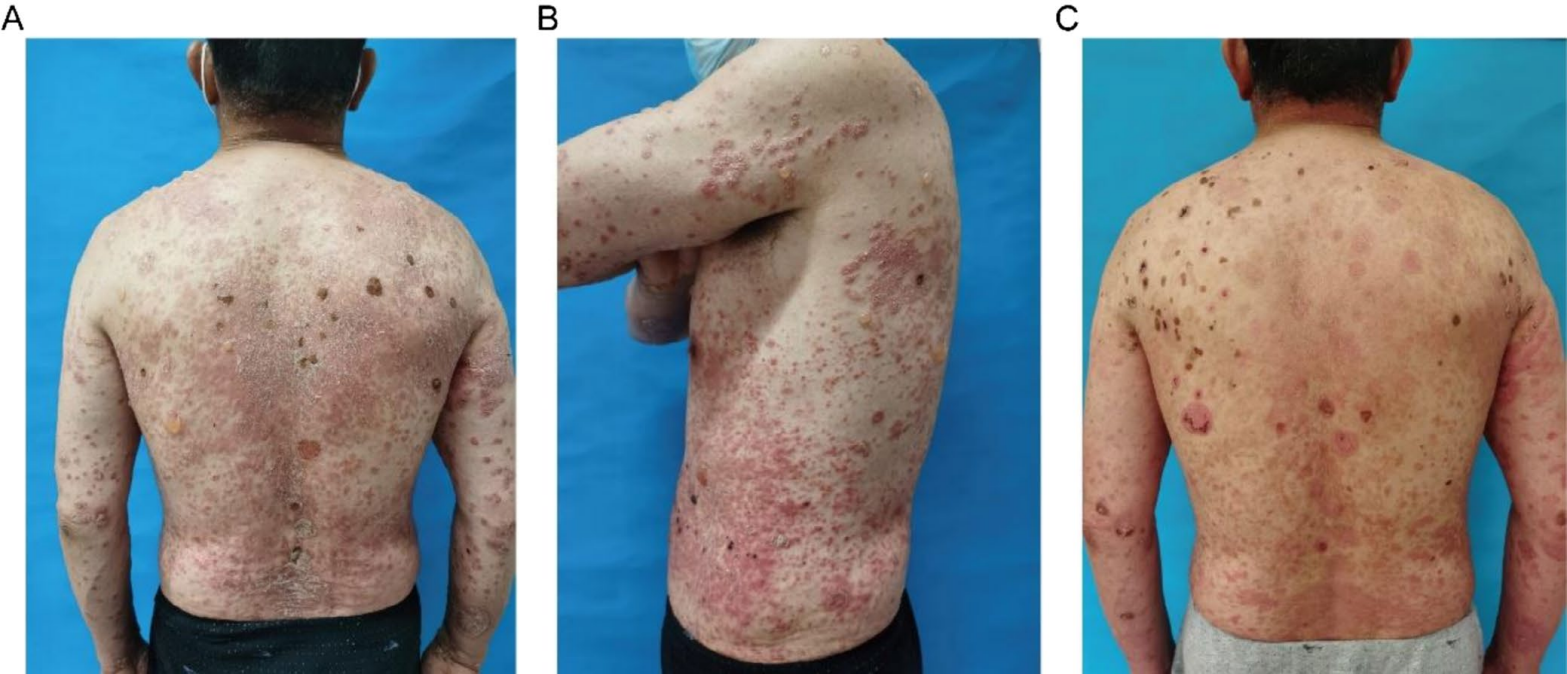
Clinical presentation before and after treatment. (A, B) Pretreatment images showing psoriatic plaques with silvery scales and multiple tense bullae on both lesional and nonlesional skin, with some erosions and crusting. (C) Posttreatment image demonstrating marked resolution of erythema, scaling, and complete healing of bullae.

The patient was a 64‐year‐old man with a 30‐year history of psoriasis vulgaris, initially diagnosed by histopathology after scaly erythematous plaques appeared on the bilateral lower extremities without identifiable triggers. He managed the condition with intermittent use of unspecified traditional Chinese medicine and thermal spring baths, experiencing recurrent flares. Ten days before admission, his psoriatic lesions worsened abruptly, with erythematous plaques expanding to cover 60% body surface area (BSA). Five days prior to admission, approximately 15 tense bullae (0.5–2 cm diameter) emerged on both pre‐existing psoriatic lesions and unaffected skin; some bullae ruptured, forming erosions (Figure 1A,B). He reported mild pruritus but no fever, mucosal involvement, or drug allergy history.

## 2 Admission Diagnosis

- Psoriasis vulgaris.
- Bullous pemphigoid.

## 3 Physical Examination

Systemic examination revealed no abnormalities. Dermatological assessment demonstrated:

- Diffuse infiltrative erythema with multilayered silvery‐white scales on the trunk and extremities (affecting 60% BSA), exhibiting a positive Auspitz sign.
- Fifteen tense bullae (0.5–2 cm diameter) distributed on erythematous and unaffected skin, containing clear fluid.
- Nikolsky sign: Positive at perilesional sites; negative in distant unaffected areas.
- Mucosal examination: No erosions on oral or genital mucosa.

## 4 Auxiliary Examinations

### 4.1 Histopathology

- Psoriatic lesion (from the erythema of the left lower extremity): The findings demonstrated hyperkeratosis with parakeratosis, elongated rete ridges, and tortuous dilated capillaries in the dermal papillae, which were consistent with the diagnostic features of psoriasis.
- Bullous lesion (from fresh blisters on the back): Histopathological examination revealed subepidermal blister formation, along with a marked dermal inflammatory infiltrate predominantly consisting of eosinophils and neutrophils.

### 4.2 Direct Immunofluorescence of Perilesional Skin

- Continuous linear deposition of IgG (strong intensity, ++) along the basement membrane zone.
- Granular deposition of C3 (moderate intensity, +).
- Focal weak positivity for IgA.

### 4.3 Other Tests

Complete blood count showed mild eosinophilia (0.54 × 10^9^/L). Supportive serological findings are summarized in Table 1. These include elevated inflammatory markers consistent with disease activity, significantly reduced IgG levels, and a negative comprehensive autoantibody panel, which collectively aided in supporting the diagnosis of BP and excluding other autoimmune blistering disorders. Liver and renal function tests, as well as chest imaging, revealed no abnormalities.

**TABLE 1:** Inflammatory and immunological profile at admission.

Parameter	Patient value	Reference range	Unit
Inflammatory markers			
Interleukin‐6 (IL‐6)	17.7	< 7	pg/mL
C‐reactive protein (CRP)	0.912	< 0.8	mg/dL
Erythrocyte sedimentation rate (ESR)	16	0–15	mm/h
Immunoglobulins and complement			
IgG	511	860–1740	mg/dL
IgA	66.8	100–240	mg/dL
IgM	48.1	50–280	mg/dL
Complement C3	91	70–140	mg/dL
Complement C4	24.1	10–40	mg/dL
Autoantibodies			
Antinuclear antibodies (ANA)	Negative	Negative (< 1:80)	—
Anti‐dsDNA antibody	Negative	Negative (< 1:101)	—
Extractable nuclear antigen (ENA) panel	All negative	Negative (< 1:101)	—

## 5 Treatment Course

- Initial Regimen (Days 1–4)

Methylprednisolone 0.7 mg/kg/day (56 mg/day) orally in divided doses.

Nicotinamide 600 mg orally three times daily.

Topical therapy:

○ Halometasone cream is applied to erythematous areas twice daily.
○ Compound polymyxin B ointment applied to erosions twice daily.

- Modified Regimen (From Day 5 Onward)

Methotrexate 7.5 mg weekly was added, administered as 2.5 mg every 12 h for three doses.

Concurrent folic acid supplementation 5 mg weekly.

## 6 Therapeutic Outcome Assessment Standards and Follow‐Up

The Psoriasis Area and Severity Index (PASI) decreased from 18.6 at admission to 5.2 at discharge, while the Bullous Pemphigoid Disease Area Index (BPDAI) also improved from 28.6 at admission to 9.2 at discharge (Figure 1C).

Monthly outpatient reviews over 3 months post‐discharge revealed no significant recurrence of psoriasis vulgaris or bullous pemphigoid, with normal monthly laboratory monitoring (complete blood count and liver function tests). A subsequent telephone follow‐up at 6 months indicated sustained remission of bullous pemphigoid (no new blisters), although occasional small psoriatic lesions appeared on the extremities.

## 7 Discussion

This case underscores the diagnostic and therapeutic challenges when bullous pemphigoid (BP) emerges in a patient with longstanding psoriasis. The acute development of tense bullae amidst chronic psoriatic plaques (Figure 1A,B), as observed here, is a distinctive feature that heightens clinical suspicion for this specific comorbidity over other blistering disorders [1, 2]. Compared to previously reported cases, this report provides a detailed account of disease onset and management in a patient with a decades‐long history of psoriasis, adding to the literature on the clinical progression of this overlap syndrome [3].

The pathogenesis in this patient may be explained by immune crosstalk initiated within chronic psoriatic skin [4]. The sequential onset—psoriasis, followed by BP—points to a shared yet dysregulated immunology. Psoriasis is driven by Th17 cells and IL‐17, while BP is characterized by Th2 responses and anti‐BP180 antibodies [5, 6]. The distinct immune profiles, spanning key cells, cytokines, and effector molecules in each disease, are summarized for comparison in Table 2. Two nonmutually exclusive mechanisms may be particularly relevant to our patient's timeline: (1) Epitope spreading triggered by chronic inflammation, where the persistent psoriatic plaque environment could expose neoantigens like BP180, breaking tolerance and initiating an antibody response—a process supported by models of repeated skin injury [7, 8]; and (2) Cytokine‐mediated cross‐talk, where psoriatic IL‐17 may upregulate MMP‐9 from keratinocytes [9], potentially damaging the basement membrane and exposing BP180 antigens [10, 11]. The patient's prior use of unspecified traditional Chinese medicine introduces a theoretical, though speculative, possibility of treatment‐related immune modulation [12, 13, 14].

**TABLE 2:** Comparison of potential mechanisms of psoriasis combined with BP.

Mechanism	Dominant factors in psoriasis	Dominant factors in BP	Cross‐interaction node
Key cells	Th17, KC	Th2, B cells	Langerhans cell antigen presentation
Characteristic cytokines	IL‐17A, IL‐23	IL‐4, IL‐5, IL‐13	TNF‐α promotes inflammatory amplification
Effector molecules	β‐defensins, S100 proteins	Anti‐BP180 IgG, complement	MMP‐9 mediates tissue damage
Therapeutic targets	IL‐23 inhibitors	CD20 monoclonal antibodies	JAK/STAT pathway regulation

Our treatment rationale centered on rapidly controlling blistering while addressing the underlying immune dysregulation of both diseases [15, 16]. Systemic glucocorticoids provided immediate suppression [17]. Methotrexate was chosen as the steroid‐sparing agent not only for its dual immunomodulatory potential to modulate the Th17 pathway in psoriasis and suppress B‐cell responses in BP, but also to reduce the cumulative dose and long‐term toxicity of glucocorticoids [18, 19]. This combination halted new blister formation within days and significantly improved both PASI and BPDAI scores. Although biologics like anti‐IL‐17 agents are potent for psoriasis, conventional immunosuppressants were preferred in this acute BP case due to their more predictable and established role, given that the evidence for biologics in this specific overlap condition is still emerging and inconsistent [14, 20]. Vigilant monitoring for infection, hepatotoxicity, and myelosuppression was an integral part of the management protocol.

The limitations of our report should be noted. First, the absence of serial anti‐BP180/230 antibody titers not only precluded detailed immunological monitoring of disease activity but also limited our ability to fully exclude other rare autoimmune blistering diseases, such as bullous systemic lupus erythematosus, through comprehensive serological profiling. Second, both psoriasis and BP are chronic, relapsing conditions; therefore, the relatively short 6 month follow‐up period, while demonstrating promising initial control, is insufficient to assess long‐term treatment durability, relapse patterns, and potential late adverse effects. Despite these constraints, the sustained remission observed during the follow‐up period is notable. In conclusion, this case highlights that glucocorticoids combined with methotrexate can serve as an effective first‐line strategy for psoriasis patients who develop BP. It reinforces the need for vigilance, a treatment approach that targets the distinct immunologic components of both diseases, and commitment to long‐term monitoring.

## Author Contributions

**Weimin Ma:** conceptualization. **Cheng Li:** data curation, writing – original draft. **Haijun Miao:** writing – review and editing.

## Funding

The authors have nothing to report.

## Ethics Statement

This case study was approved by the Scientific Research Ethics Committee of The 940th Hospital of Joint Logistic Support Force of PLA in accordance with the ethical standards of the institutional committee and the 1964 Helsinki Declaration. The committee waived the requirement for formal ethical approval for this retrospective case report.

## Consent

Written informed consent was obtained from the patient for both participation in the study and for the publication of this case report and any accompanying images.

## Conflicts of Interest

The authors declare no conflicts of interest.

## Data Availability

De‐identified case data available from corresponding author upon reasonable request.
